# Caesarean sections among east African refugees and their host population: a 20-year retrospective study in western Tanzania

**DOI:** 10.4314/ahs.v24i4.27

**Published:** 2024-12

**Authors:** Sarah Rapaport, Hilary Ngude, Chi Chiung Grace Chen, Mohamed Abbas, Amber Lekey, Peter J Winch, Joseph V Sakran, Kent A Stevens, Zachary Obinna Enumah

**Affiliations:** 1 Global Surgery Initiative, Department of Surgery, Johns Hopkins Hospital, Baltimore, MD, USA; 2 Tanzania Red Cross Society, Dar es Salaam, Tanzania; 3 Department of Gynecology and Obstetrics, Johns Hopkins University School of Medicine, Baltimore, MD, USA; 4 Department of Surgery, Tulane University School of Medicine, New Orleans, LA, USA; 5 Department of International Health, Johns Hopkins School of Public Health, Baltimore, MD, USA; 6 Satcher Health Leadership Institute, Morehouse School of Medicine, Atlanta, GA, USA

**Keywords:** Refugee health, caesarean section, humanitarian setting, global surgery, Tanzania

## Abstract

**Background:**

Half of the 35.3 million refugees worldwide are women and a third are of childbearing age, making reproductive healthcare in humanitarian settings indispensable. Caesarean section (CS) is the most common operation worldwide, accounting for 18.6% of births.

**Objective:**

This study provides a descriptive analysis of caesarean sections (CS) in a protracted refugee setting.

**Methods:**

This study is a retrospective review of CS recorded in logbooks between November 2000 and September 2020, inclusive. Data was abstracted from paper logbooks and included date, nationality, sex, age, and indication. Analysis was performed in STATA.

**Results:**

8,461 CS were performed over the 20-year period. The average age was 24.6 years. Tanzanian patients made up 25% (n=2,116) of the population. The most common indications for CS for both Tanzanians and refugees were cephalopelvic disproportion (CPD), previous scar, and fetal distress. There was a significant difference in the proportion of adolescent and adult patients for the indications of CPD, previous scar, fetal distress, malposition, placenta previa, prolapse, and antepartum hemorrhage (APH).

**Conclusion:**

There is a significant amount of CS performed in this humanitarian setting. Refugees and Tanzanians utilize these services. Outcome data are needed to identify if CS in this setting reduces neonatal and maternal morbidity and mortality.

**Funding:**

American Society of Tropical Medicine and Hygiene; Ruth L. Kirschstein National Research Service Award T32 (Award: 2T32AR67708-6); Association for Academic Surgery

## Introduction

Over 108.4 million people worldwide were forced to flee their homes with nearly 35.3 million classified as refugees, measured at the end of 2022^1^. About 22% of refugees reside in camps, which provide housing, food, and healthcare^2^. Refugees have complex health needs, including the triple burden of infectious disease, mental health needs, and non-communicable diseases^3^. Providing healthcare for refugees is challenging, which is complicated by the location of camps in resource limited settings and that humanitarian settings are often impacted by chronic underfunding^1^,^4^,^5^. Reproductive healthcare is a critical component of refugee camp medicine, as women between the ages of 12-59 make up a third of forcibly displaced persons^6^.

Caesarean sections (CS) are the most commonly performed operation globally. In 2015, it was estimated that 29.7 million CS were performed that year^7^,^8^. The goal of a CS is to reduce maternal and neonatal morbidity and mortality during complicated births. Access to CS care is implicated in the Sustainable Development Goals, particularly SDG^3^, which by 2030 aims to decrease maternal mortality to 70/100,000 and ensure universal reproductive healthcare worldwide^9^. There is debate about the optimal percent of CS births, but the WHO declares 10-15% as the ideal range between over and underutilization^10^. Under 10% CS utilization is associated with increases in maternal and neonatal morbidity and mortality, while over 15% increases the likelihood of maternal complications, as absolute maternal mortality is higher for CS than for vaginal births^10^–^13^. Despite increases in CS rates worldwide and estimates showing a doubling in the number of CS performed between 2000 and 2015, many locations lack access to safe and timely CS^8^. Low-income settings, particularly in sub-Saharan Africa, have the least access to these potentially lifesaving operations, with many areas falling below the 10% threshold^14^,^15^.

Studies on CS among refugee populations in sub-Saharan Africa compare CS access between refugee camps and the surrounding population, finding that when host communities fall below the 10% critical value, refugee camps provide increased access for both refugees and host communities^16^–^18^. To our knowledge, no group has conducted a longitudinal study of this size assessing CS among refugees in Tanzania or CS among adolescent refugees in sub-Saharan Africa, while only few studies exist assessing CS among adolescents in sub-Saharan Africa. The objective of our study is to provide a descriptive analysis of CS in a protracted humanitarian setting over a twenty-year period in one of the largest refugee camps in sub-Saharan Africa.

## Methods

### Study setting and population

Nyarugusu Camp is located in Kigoma, western Tanzania. It is the largest refugee camp in Tanzania. Originally founded in 1996 for refugees from the Democratic Republic of Congo, it is now additionally home to refugees from Burundi. Its current population is about 133,000 with a population breakdown of 51% female (49% male) and 55% children under the age of 17, with 27% of the camp population females under 17 years of age^19^,^20^.

The Tanzania Ministry of Home Affairs and the United Nations High Commissioner for Refugees (UNHCR) are responsible for provision of services and administration in the camp, although non-governmental (NGOs) and multilateral organizations provide additional operational support. Medical services within the camp are coordinated by the Tanzanian Red Cross Society (TRCS). The main medical services include a dispensary hospital (where CS operations occur), two health centers, and many health posts. Tanzanians can access the camp health services in addition to the refugees the camp primarily serves. Services at the camp are provided at no cost to both refugees and Tanzanians, which differs from local Tanzanian district hospitals. Cases requiring care beyond the scope that the camp can provide are referred according to UNHCR referral guidelines and practices^21^.

At the main camp dispensary hospital, there are two major operating rooms and one minor operating room. Procedures performed in the major operating rooms are recorded by camp staff in paper logbooks. The camp has a dedicated maternity ward staffed by dedicated maternity nurses and camp physicians. There is limited access to peripartum monitoring technology, with camp staff mainly using a pinard horn and ultrasound. When a patient has an indication for a CS, they undergo the operation in one of the major operating rooms by the provider on call, which is documented in the logbook. The healthcare workers performing CS are the same as those performing other general surgery operations. These providers have typically completed medical school and one intern year. In the antepartum and postpartum setting, there is a very busy reproductive and child health clinic that serves children under five.

### Data collection

A retrospective review of paper surgery logbooks in Nyarugusu Camp was conducted. Operations occurred between November 2000 and September 2020, inclusive. The following data were abstracted for analysis: date of procedure, patient nationality, sex, age, and indication for CS. Data that were abstracted but not included for analysis include time of procedure, post-operative diagnosis, and Apgar score. These variables were excluded due to inconsistent recording in the logbook. Only procedures coded as caesarean sections were included for analysis. Outcome data was a single column in the logbook and was not collected for analysis because it was inconsistently recorded and only descriptive (“good”, “fair”). These handwritten logbook entries were digitized into a Microsoft Excel spreadsheet. A second team member checked 10% of entries for quality control. If any part of an entry could not be read by either reviewer, it was marked as illegible.

### Statistical analysis

Each caesarean section operation could include between one and three indications and one and three kinds of anesthesia. Both the indication for CS and type of anesthesia were standardized for analysis. Because data were de-identified, the total number of procedures is not representative of unique patients, as more than one caesarean section could have been performed on a single patient. Patient nationality, age, sex, indication, and anesthesia used underwent descriptive analysis. Analysis was performed using STATA statistical software (Version 16. StataCorp; College Station, TX).

To determine a rate of CS among the population, we utilized monthly reports from the year 2018 (as an example of recent year) that provide data on total deliveries in the refugee camp. Data was missing for August and September 2018. Despite these two months missing for the year 2018, this year was chosen as it was the most recent year that had the most available data – accessible reports from other years were missing more than two months and/or were not as recent. We computed a CS rate by utilizing the number of CS in our study population over those ten months as the numerator, with a denominator of total deliveries from the monthly reports.

### Ethics approval

This study was approved by the Tanzanian Commission on Science and Technology (COSTECH) (2020-391-NA-2011-143) and the Johns Hopkins Institutional Review Board (IRB00012663). The Johns Hopkins Institutional Review Board waived informed consent. All procedures and processes were conducted according to applicable regulations and guidelines.

## Results

### Demographics

**Figure 1:**
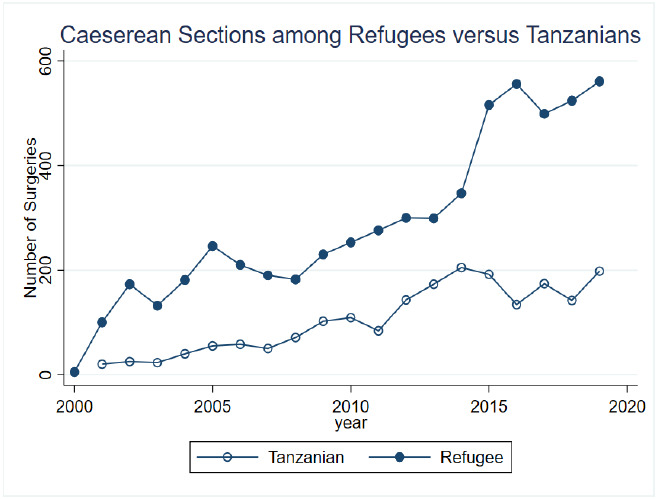
Caesarean sections in refugees versus host population over time

A total of 8,461 caesarean section operations were performed over the 20-year study period. There was a steady increase in caesarean sections performed over the four, five-year time periods, with 1,041 operations performed between November 2000-2005, 1,463 between 2006-2010, 2,571 between 2011-2015, and 3,386 between 2016 and September 2020 (Figure 1). The average age over the 20-year period was 24.6 years (SD 6.9) and this remained constant throughout the study period. Individuals between the ages of 18-24 made up the largest proportion of patients (n=3,855; 46%), followed by patients between the ages of 25-29 (n=1,557;18%) and those between 30-34 (n=1,107; 13%). Patients of advanced maternal age, defined as 35 and older, made up 11% (n=926) of the population, while adolescent patients under the age of 18 comprised 10% (n=10%) of the sample. There was variability among the most represented age categories over the twenty years, most notably with the proportion of adolescent patients steadily decreasing (15% in 2000-2005, 14% in 2006-2010, 12% in 2011-2015, and 6% in 2016-2020) (Table 1).

**Table 1:** Demographics over time

	Overall 2000-2020	2000-2005	2006-2010	2011-2015	2016-2020
N	8,461	1,041	1,463	2,571	3,386

Average age (SD)	24.6 (6.9)	24.2 (7.1)	24.7 (7.4)	24.4 (7.1)	24.8 (6.4)

Age (in years)					
<18	878 (10.4%)	160 (15.4%)	205 (14.0%)	318 (12.4%)	195 (5.8%)
18-24	3,855 (45.6%)	421 (40.4%)	593 (40.5%)	1,170 (45.5%)	1,671 (49.4%)
25-29	1,557 (18.4%)	168 (16.1%)	243 (16.6%)	421 (16.4%)	725 (21.4%)
30-34	1,107 (13.1%)	133 (12.8%)	211 (14.4%)	344 (13.4%)	419 (12.4%)
35+	926 (10.9%)	106 (10.2%)	193 (13.2%)	303 (11.8%)	324 (9.6%)
Missing	138 (1.6%)	53 (5.1%)	18 (1.2%)	15 (0.6%)	52 (1.5%)

Nationality					
DRC	4,544 (53.7%)	832 (79.9%)	1,065 (72.8%)	1,441 (56.0%)	1,206 (35.6%)
Burundi	1,464 (17.3%)	0 (0.0%)	0 (0.0%)	231 (9.0%)	1,233 (36.4%)
Tanzanian	2,116 (25.0%)	163 (15.7%)	390 (26.7%)	797 (31.0%)	766 (22.6%)
Other	134 (1.6%)	6 (0.6%)	1 (0.1%)	67 (2.6%)	60 (1.8%)
Missing	203 (2.4%)	40 (3.8%)	7 (0.5%)	35 (1.4%)	121 (3.6%)

Tanzanian patients utilizing the humanitarian hospital services made up 25% (n=2,116) of the population. Among refugee patients, those from the Democratic Republic of Congo (DRC) comprised 53.7% (n=4,544) of patients, followed by those from Burundi (n=1,464; 17%), and “other” (n=134; 2%), which includes patients from Rwanda, Kenya, and those recorded as “refugee” in the logbook. There was variability in the proportion of nationalities over the twenty-year period, most notably with a decrease in the proportion of refugees from DRC and an increase in those from Burundi. This is explained in part by the camp primarily serving those from DRC until 2015 when an influx of Burundian refugees arrived. The proportion of Tanzanians utilizing humanitarian services was also variable but with no identifiable trend (Table 1).

### Indications for caesarean sections among Tanzanians and refugees

After eliminating entries with no indication or nationality recorded in the logbook, there remained 8,267 patients, 26% (n=2,119) of whom were Tanzanian and 74% (n=6,148) were refugees. The most common indications for CS for the total study population (combined Tanzanians and refugees) were cephalopelvic disproportion (CPD) (n=3,780; 47%), previous scar (n=2,191; 27%), fetal distress (n=1,372; 17%), malposition (n=1,132; 17%), and placenta previa (n=251; 3%). Tanzanians were more likely to undergo CS for CPD (p<0.001) and placenta previa (p=0.013) while refugee women were more likely to undergo CS for scar (p=0.001) and malposition (p=0.027) (Table 2).

**Table 2:** Common indications for caesarean section by host population and refugee status

	Total	Tanzanian	Refugee	P-value
N	8,267	2,119 (25.6%)	6,148 (74.4%)	

CPD	3,780 (46.6%)	1,032 (49.9%)	2,748 (45.5%)	<0.001*
Scar	2,191 (27.0%)	504 (24.3%)	1,687 (28.0%)	0.001*
Fetal Distress	1,372 (16.9%)	340 (16.4%)	1,032 (17.1%)	0.48
Malposition	1,132 (14.0%)	259 (12.5%)	873 (14.5%)	0.027*
Placenta Previa	251 (3.1%)	81 (3.9%)	170 (2.8%)	0.013*
Prolapse	195 (2.4%)	53 (2.6%)	142 (2.4%)	0.60
APH	108 (1.3%)	28 (1.4%)	80 (1.3%)	0.93
Eclampsia	80 (1.0%)	15 (0.7%)	65 (1.1%)	0.16
Uterine Rupture	65 (0.8%)	18 (0.9%)	47 (0.8%)	0.69
Maternal Distress	55 (0.7%)	11 (0.5%)	44 (0.7%)	0.34

* Indicates statistically significant P-value

** Prolapse is a broadly coded category that includes uterine, vaginal, etc. and eclampsia represents any condition on the eclampsia spectrum including pre-eclampsia

*** CPD= cephalopelvic disproportion; scar= previous scar; APH= antepartum hemorrhage

### Indications for caesarean section between adolescent and adult patients

After eliminating entries with no age or indication recorded in the logbook, there remained 8,476 patients. Ten percent (n=879) were adolescents under the age of 18 while 90% (n=7,597) were adults aged 18 years and older. Among the total study population that included both Tanzanians and refugees, there were differences in the top five indications for CS between adolescents and adults. Among adolescents, the top five indications for CS were CPD (n=532; 61%), fetal distress (n=179; 21%), malposition (n=97; 11%), previous scar (n=35; 4%), and eclampsia (n=13; 2%). Among adults, the top five indications were CPD (n=3,333; 45%), previous scar (n=2,234; 30%), fetal distress (n=1,219; 16%), malposition (n=1,055; 14%), and placenta previa (n=249; 3%). Adolescent patients were more likely to undergo CS for CPD (p<0.001) and fetal distress (p=0.002), while adult patients were more likely to undergo CS for previous scar (p<0.001), malposition (p=0.027), placenta previa (p<0.001), prolapse (p<0.001), and antepartum hemorrhage (APH) (p=0.018) (Table 3).

### Caesarean section rate

In the ten months of health report data available from the Tanzania Red Cross for 2018, there were 5612 deliveries. Our data noted 560 CS over these ten months. Thus, the estimated caesarean section rate in this setting is 9.98%.

## Discussion

### Main findings

To our knowledge, this is the largest dataset on CS among refugees in sub-Saharan Africa. Nyarugusu Camp is the largest humanitarian setting in Tanzania and one of the largest in the world, making this data a valuable contribution to the literature on CS in humanitarian settings. Our data shows there were 8,461 CS performed over the 20-year period, with a steady increase in operations performed between 2000 and 2020. There was a decrease in adolescent CS over the twenty-year period. The most common indications for CS among the total population of refugees and Tanzanians were CPD, previous scar, and fetal distress. CPD, fetal distress, and malposition were the most common indications among adolescents. Adolescents were more likely than adults to undergo CS for CPD and fetal distress, while adults were more likely to undergo CS for previous scar, malposition, placenta previa, prolapse, and antepartum hemorrhage.

### Caesarean section rate

Our calculated CS rate for the year 2018 was 9.98%. In light of the ideal rate of CS declared by the WHO to be 10-15% and previous research showing that CS rates below 10% are correlated with higher maternal and neonatal mortality and morbidity, this estimated rate is at the cusp of appropriate utilization^10^,^11^. Research from 2006 stratifying CS access by low-, middle- and high-income countries show that 76% of low income, 16% of middle income, and 3% of high-income countries have CS rates 10% or under and that only low-income countries, not middle- and high-income countries, have a negative correlation between CS rates and both neonatal and maternal mortality^12^.

Research from 2015-2016 found the CS rate in Tanzania to be 6%. If the assumption holds that the CS rate in Tanzania did not significantly increase from 2016 to 2018, and that the rate in the camp in 2016 is similar to that in 2018, this data reveals that patients from the camp and surrounding community have better access to CS care than the average Tanzanian^22^. However, the lack of outcome data in our dataset prohibits our ability to state the appropriateness of the 9.98% rate for the setting and highlights the importance of outcome data in determining the ideal CS rate for a setting. The lack of surgical outcome data is a common limitation in humanitarian settings, yet is crucial for improving the quality of care^23^.

### Trends in number of caesarean sections over time

There was a steady increase in caesarean sections in Nyarugusu Camp over the twenty-year study period, with over three times the number performed between 2016-2020 compared to 2000-2005. Worldwide, there was also an increase in caesarean sections during this time, with almost double the number of caesarean sections performed in 2015 compared to 2008. In Tanzania, between 1996 and 2016, there was a fivefold increase in CS. This increase in CS is hypothesized to have occurred due to increasing resources to perform such operations, including widespread access to electricity, anesthesia availability, and 24 hour availability of providers^22^. There are several reasons that could explain the increase in CS over time in the camp. First, there was an increase in capacity to perform caesarean section, due to increased access to anesthesia, and skilled labor. Additionally, there could be a snowball type effect: as more women had access to caesarean sections in the camp in the early 2000s, these women had caesarean sections for future pregnancies, which would increase total numbers of caesarean sections when combined with nulliparous women also having a caesarean section. Unfortunately, without outcome data, which are rarely able to be collected in humanitarian settings, we are not able to comment on the appropriateness of the increased number of caesarean sections, or if this increased number sufficiently improved or is meeting a critical need. However, one thing is for certain: CS in adolescents carries important implications for their future birthing options and reproductive healthcare.

Across studies, the common indications for CS among adolescents include low birth weight, prematurity, early neonatal death, and pre-eclampsia/eclampsia^33^–^36^. The adverse events leading to CS among these studies appear to be primarily fetal, which contradicts the literature among adults that finds indications for CS to be primarily maternal. However, these findings mirror those of our study in fetal distress being the second most common indication and eclampsia being one of the most common among adolescents. Further research is needed among pregnant adolescents and the indications that lead to CS in both refugee and local populations.

## Limitations

Our study is not without limitations. The transient nature of camp staff combined with a lack of a standardized recording system adds heterogeneity and subjectivity into the originally recorded data. Inconsistencies in recorded variables (such as Apgar scores only rarely recorded) provided only limited variables. Data on the emergent nature of procedures and outcomes were lacking. Additionally, in calculating our CS rate, we did not have data on total births for August and September 2018.

## Conclusion

There is a substantial number of caesarean sections performed in one of the largest humanitarian settings in the world, and like the rest of the world, this number has increased between 2000 and 2020. Despite this increase in overall CS, a decrease in CS among adolescents possibly reveals advancements in access to reproductive healthcare among encamped adolescent refugees and local adolescents using the camp healthcare services. While there was a large number of caesarean sections performed during the study period, in 2018, one of the later years of the study, the CS rate was just below the 10% cutoff of appropriate utilization. While this work elucidates the capacity of a large humanitarian setting to provide an integral component of healthcare, the lack of outcome data limit our ability to ascertain the appropriateness of CS care and if this care is sufficiently meeting the need. We hope this work will aid in informing future endeavors to quantify outcome data on CS in humanitarian settings as well as future capacity building efforts for improving CS care.

## Data Availability

Data used and analyzed in the current study are not publicly available due to privacy and personally identifiable health information. De-identified, aggregate data is available from corresponding author upon request.

## Declarations

Ethics Approval and Consent to Participate – The study was approved by the Johns Hopkins Institutional Review Board and Tanzanian Commission on Technology and Science.
